# Supplementary material for: Ultrafast zero balance of the oscillator-strength sum rule in graphene
Source: Sci Rep. 2013 Sep 16;3:2663. doi: 10.1038/srep02663 (PMC3773626; doi:10.1038/srep02663)
Supplement: Supplementary Information [file srep02663-s1.doc]

Supplementary Information for “Ultrafast zero balance of the oscillator-strength sum rule in graphene”

Jaeseok Kim^1†^, Seong Chu Lim^2,3†^, Seung Jin Chae^2,3^, Inhee Maeng^1^, Younghwan Choi^1^, Soonyoung Cha^1^, Young Hee Lee^2,3*^ and Hyunyong Choi^1*^

*^1^ School of Electrical and Electronic Engineering, Yonsei University, Seoul 120-749, Korea*

*^2^ IBS Center for Integrated Nanostructure Physics, Institute for Basic Science, Sungkyunkwan University, Suwon, 440-746, Korea*

*^3^ Department of Energy Science, Department of Physics, Sungkyunkwan University, Suwon 440-746, Korea*

^*^e-mail: leeyoung@skku.edu (Y.H.L.); hychoi@yonsei.ac.kr (H.C.)

^†^These authors contribute equally to this work.

**Characterization of the single-layer graphene**

The crystallinity and morphology of the single-layer graphene were characterized using Raman spectroscopy and atomic force microscopy (AFM). In Raman spectroscopy excited by a 532 nm laser beam, the strong 2D-band located at 2694 cm^-1^ and the highly symmetric Lorentzian curve clearly indicated the presence of single-layer graphene (Fig. S1a). In addition, the high intensity ratio of the G-band (1590 cm^-1^) to the D-band (1350 cm^-1^) is evidence for high crystallinity in the layer of our graphene sample. The morphology of graphene was examined using AFM. The graphene exhibited numerous wrinkles and grain boundaries, as illustrated in Fig. S1b. The electrical characterization of the graphene was performed on a diamond substrate. The I–V measurements showed ohmic behavior, as illustrated in Fig. S1c. Furthermore, we performed a Hall measurement to determine the carrier type and concentration. The graphene was a *p*-type conductor with a hole carrier concentration of *P*_0_ = 6.88 × 10^12^ cm^-2^. The Fermi energy of graphene was then estimated to be *E*_F_ ~ -*ħv*_F_$\sqrt{\pi P_{0}}$= -306 ± 5 meV, with a graphene Fermi velocity of *v*_F_ = 1 × 10^6^ m/s.

**
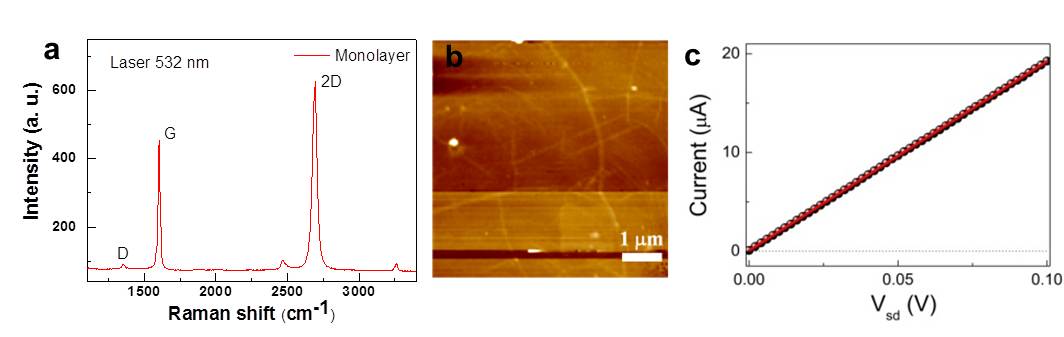
**

**Figure S1.** **a**, Raman spectrum and **b**, surface morphology characterized using AFM. **c**, I–V curve of the single-layer graphene.

**Quasi-Fermi level changes**

Figure S2 show the ultrafast response near the hot-Fermi tail performed by all-optical pump-probe measurements Δ*T*/*T*_0_ with a much higher time resolution (~ 50 fs) than that of the THz probe (~ 1 ps). By comparing the all-optical Δ*T*/*T*_0_ with the THz Δ*E*/*E*_0_ dynamics, it is clear that Δ*E*/*E*_0_ contains a delayed population decrease (Δ*t_d_* ≈ 0.35 ps) compared to the all-optical measurements. The difference in Δ*t_d_* reflects the fact that there is an ultrafast energy dissipation process during Δ*t* (rapid changes of quasi-Fermi level) caused by the decay component *τ*_1_ ≈ 300 fs, which was not captured in the THz measurements. We define this additional loss constant as *β* (= exp(-Δ*t_d_* /*τ*_1_)), which has a value of 0.3 in our case.


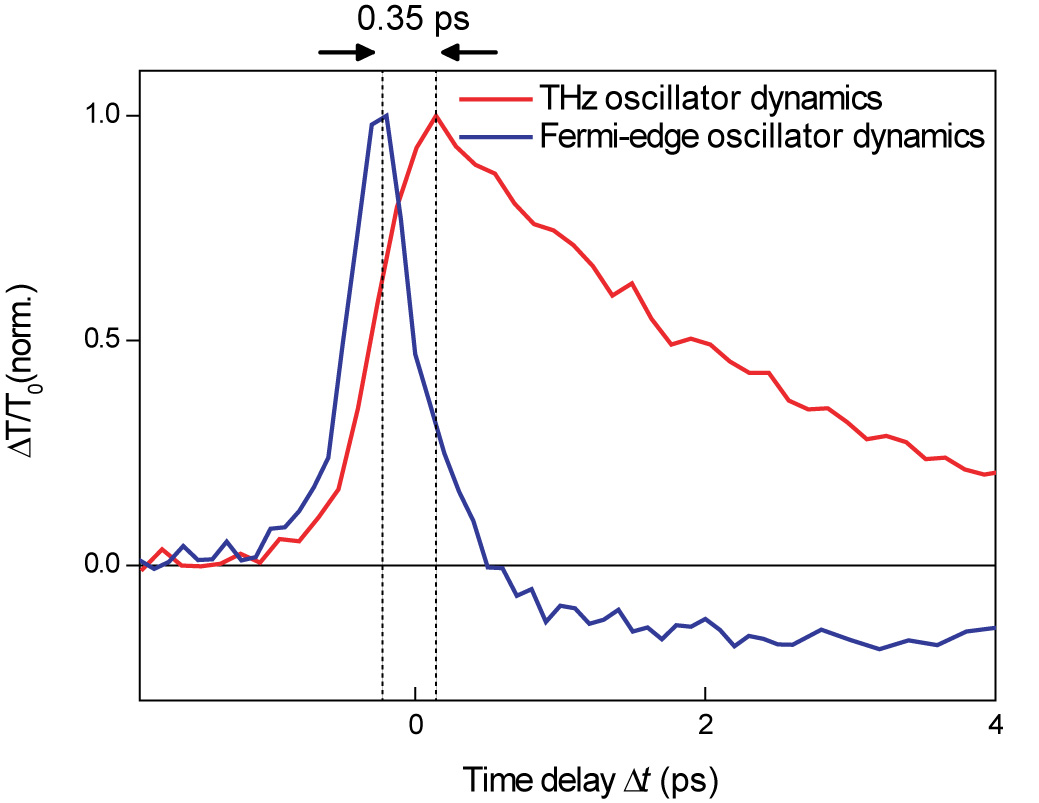


**Figure S2.** The THz oscillator dynamics (red line) and the Fermi-edge oscillator dynamics near the Fermi-level of 620 meV (blue line). The experimental conditions (pump-photon energy of 1.55 eV and pump fluence of 40 µJ/cm^2^) are same as in the main text.

In doped graphene, the Fermi level is defined by^1^

for $k_{B}T<<E_{F}$. (S1)

When the pump pulse excites the graphene sample, the quasi-Fermi level changes of electrons and holes are separated. Immediately after the pump, the quasi-Fermi level change of electrons (minority carriers in *p*-type graphene) in the conduction band is

(S2)

where we include the ultrafast changes of quasi-Fermi level as shown in Fig. S2. The photo-excited electron density corresponds to Δ*n* = 0.63 × 10^12^ cm^-2^ given the excited pump fluence of 40 µJ/cm^2^ and the graphene optical absorption 2*Z*_0_*σ*_0_ / (*n*_s_ + 1) 1.3 %, where *Z*_0_ is the vacuum impedance, *σ*_0_ = *q*^2^*π* / 4*h* is the quantum conductivity, and *n*_s_ = 2.4 is the refractive index of the diamond substrate.

Similarly, the quasi Fermi level change of the holes (majority carrier in our *p*-type graphene) in the valence band is

(S3)

The photo-excited initial hole density corresponds to Δ*p* = 0.63 × 10^12^ cm^-2^ .

**Low-energy conductivity oscillators in the THz range**

In the low-energy quasi-free THz range, the graphene conductivity at equilibrium state is expressed by^2^

(S4)

where *τ* is the momentum scattering time. For the highly doped graphene with an *E*_F_ greater than the thermal excitation energy (*E*_F_ >> *k*_B_*T*), the conductivity is approximated as:

(S5)

The photo-excited conductivity changes are obtained from the difference between the non-equilibrium and equilibrium conductivities. The photo-excited hole density corresponds to Δ*p* = 0.63 × 10^12^ cm^-2^. The equation for the majority response is^3^

(S6)

For highly doped graphene, the response is dominated by the photo-excited minority carrier density Δ*n*. The equation for the minority response is^4^

(S7)

The experimental conductivity obtained by the following thin-film conductivity formula is^5^

$\begin{matrix} \Delta\sigma(\omega) =-\frac{1}{Z_{0}}(\frac{E_{0}}{E_{0}+\Delta E}-1)(n_{\text{s}}+1+Z_{0}\sigma_{0}) \\ \cong-\frac{1}{Z_{0}}(\frac{\Delta E}{E_{0}})(n_{\text{s}}+1). \end{matrix}$ (S8)

**High-energy conductivity oscillators near the Fermi edge.**

In the high-energy interband regime, the conductivity is expressed by^6^

(S9)

where *f*_e(h)_ is the electron (hole) Fermi distribution function. Again, for highly doped graphene, the conductivity is

. (S10)

The experimental conductivity is obtained via the following thin-film conductivity formula:

$\begin{matrix} \Delta\sigma_{1}(\omega) =\frac{1}{Z_{0}}(\sqrt{\frac{T_{0}}{T_{0}+\Delta T}}-1)(n_{\text{s}}+1+Z_{0}\sigma_{0}) \\ \cong\frac{1}{Z_{0}}(\sqrt{\frac{T_{0}}{T_{0}+\Delta T}}-1)(n_{\text{s}}+1) \\ =\frac{1}{Z_{0}}(\sqrt{\frac{1}{1+\Delta T/T_{0}}}-1)(n_{\text{s}}+1) \\ \cong\frac{1}{Z_{0}}(1-\frac{1}{2}\frac{\Delta T}{T_{0}})(n_{\text{s}}+1). \end{matrix}$ (S11)

For the pump fluence-dependent experiment, the transmission change is expressed as

$-\frac{\Delta T}{T_{0}}\propto\Delta\sigma(\omega)\propto\Delta E_{\text{F,h}}=E_{\text{F}}\frac{1}{2}\frac{\Delta p}{P_{0}},$ (S12)

where , and .

The photo-excited conductivity change near the Fermi edge is obtained using

$\begin{matrix} \mathrm{Re}[\Delta\sigma(\omega)]=\mathrm{Re}[\sigma_{\text{noneq}}(\omega)-\sigma_{\text{eq}}(\omega)] \\ =\mathrm{Re}[\sigma_{\text{inter}}(\omega,E_{\text{F}}+\Delta E_{\text{F,h}},T_{\text{L}}+\Delta T)-\sigma_{\text{inter}}(\omega,E_{\text{F}},T_{\text{L}})], \end{matrix}$ (S13)

where Δ*E_F,h_* is the photo-induced Fermi level change, *T*_L_ = 77 K is the lattice temperature, and Δ*T* is the photo-induced temperature change. For the same pump fluence as that used in the THz experiment, we are able to fit our data with the initial carrier temperature of *T*_L_ *+* Δ*T* = 1100 K, which is assumed to decay with bi-exponential decay components of 0.3 ps and 5.5 ps.

**THz Drude-scattering time**

The time-dependent Drude scattering time can be estimated based on the following simple proportionality relationship between the carrier density *n* and DC conductivity $\sigma_{dc}$^7^,

$\begin{matrix} \sigma_{dc}=\frac{nq^{2}\tau}{m^{*}}, \\ \frac{\tau}{m^{*}}=const. \end{matrix}$ (S14)

The effective mass $m^{*}$ in graphene is defined by ^8^. According to this simple estimation, the Drude scattering time $\tau$should be proportional to the carrier density $\tau\sim\sqrt{n}$. However, the THz measurements show an opposite behaviour, namely the measured scattering time increases as the carrier density decreases with the pump-probe delay (Table S1). This contradictory, as well explained in Ref 9, can be resolved by noting that the Drude scattering time decreases if the contribution of the pump-enhanced broadening becomes much larger than the state-filling effect.

| Time delay $\Delta t$ | 0.2 ps | 1.2 ps | 2.2 ps | 3.2 ps | 4.2 ps |
| --- | --- | --- | --- | --- | --- |
| $\tau_{eq}$ | 100 fs | 100 fs | 100 fs | 100 fs | 100 fs |
| $\tau_{noneq}$ | 88 fs | 90 fs | 92 fs | 95 fs | 100 fs |

**Table S1.** The time-dependent Drude-scattering rate is obtained by the Drude fit to the THz dynamics.

**Carrier temperature model.**

The initial carrier temperature *T*_i_ is obtained from the following energy conservation:

$U_{p}(T_{L},P_{0})+E_{a}=U_{p}(P_{0}+\Delta p,T_{i})+U_{n}(\Delta n,T_{i}),$ (S15)

where the lattice temperature *T*_L_ = 77 K and *E_a_* is the absorbed pump-pulse energy. *U*_p,n_ is the electron or hole internal energy:

(S16)

The calculated initial temperature is *T_i_* = 1100 K. However, the actual *T*_i_ (a fit to the measured data) is somewhat lower than the calculated result because of the rapid energy loss channel induced by the ultrafast optical phonon scattering (i.e., by the so-called strongly-coupled optical phonon).

**Spectrally and temporally resolved dynamics near the Fermi edge**

The dynamics near the Fermi edge (2*E*_F_ = 612 meV) represent changes in the non-equilibrium carrier occupation probability (Fig. S3a). The spectrally and temporally resolved transient dynamics near the Fermi edge are illustrated in Fig. S3b. The dynamics at 620 meV above the Fermi edge is always positive. However, the dynamics at 590 – 610 meV below the Fermi edge undergoes a sign change with increasing pump-probe delay, which implies that the reduced occupation probability below the Fermi edge transforms into an increased one due to the rapid time-dependent cooling kinetics toward an equilibrium distribution^10,11^. As the probe photon energy moves further away from the Fermi edge, the carrier occupation decays more rapidly. At each probe photon energy, the transient dynamics is fitted using a bi-exponential function (590 meV: 310 fs and 6 ps; 600 meV: 340 fs and 1.48 ps; 610 meV: 350 fs and 1.13 ps; 620 meV: 320 fs and 1.24 ps). The hot Fermi distribution tail of the probe photon energy at 590 meV has two decaying components similar to our carrier temperature model with time constants of 0.3 ps and 5.5 ps. Figure S3c shows the measured zero-crossing of equilibrium and non-equilibrium conductivity. When the pump-induced non-equilibrium distribution cools to the equilibrium distribution, the zero-crossing approaches the equilibrium Fermi level (2*E*_F_ = 612 ± 10 meV), which provides us with a self-consistency check for determining *E*_F_.


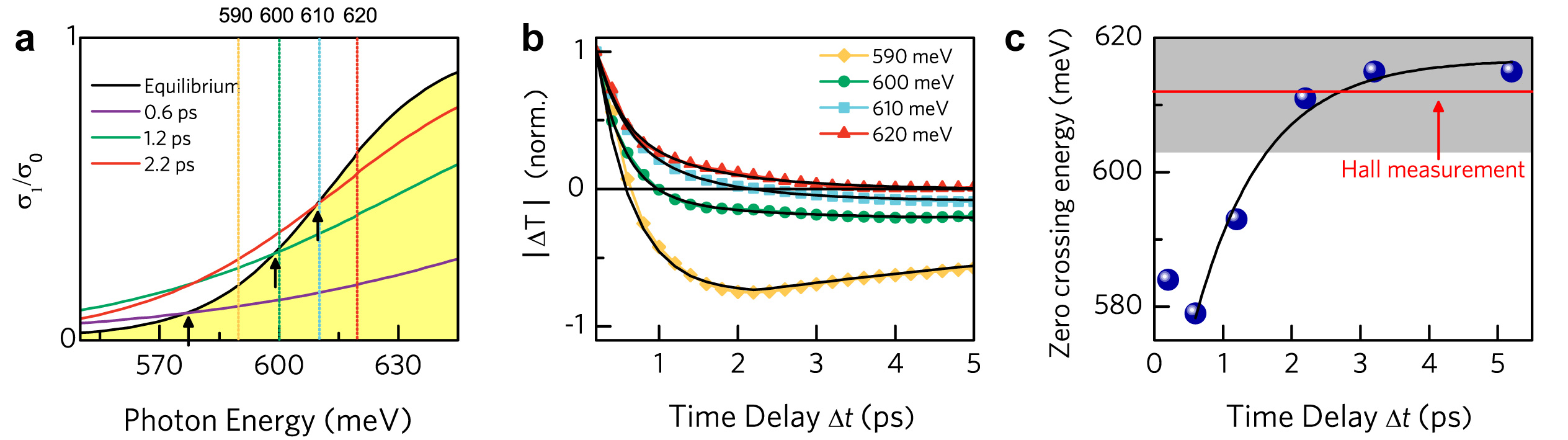


**Figure S3.** **a**, Equilibrium conductivity (black solid line) and a few representative non-equilibrium conductivities are shown for different Δ*t*. **b**, Spectrally and temporally resolved dynamics near the Fermi edge are plotted as a function of Δ*t*. **c**, The *E*_F_ measured by the Hall measurement can be double-checked using ultrafast pump-probe spectroscopy. The time-dependent shift of the zero-crossing toward 2*E*_F_ is clearly visible.

**References**

1 Hwang, E. H., Adam, S., & Das Sarma, S. Carrier transport in two-dimensional graphene layers. Phys. Rev. Lett. **98**, 186806 (2007)

2 Mikhailov, S. A. Non-linear graphene optics for terahertz applications. *Microelectron. J.* **40**, 712-715 (2009).

3 Choi, H. *et al.* Broadband electromagnetic response and ultrafast dynamics of few-layer epitaxial graphene. *Appl. Phys. Lett.* **94**, 172102 (2009).

4 George, P. A. *et al.* Ultrafast optical-pump terahertz-probe spectroscopy of the carrier relaxation and recombination dynamics in epitaxial graphene. *Nano Lett.* **8**, 4248-4251 (2008).

5 Kaindl, R. A., Hägele, D., Carnahan, M. A. & Chemla, D. S. Transient terahertz spectroscopy of excitons and unbound carriers in quasi-two-dimensional electron-hole gases. *Phys. Rev. B* **79**, 045320 (2009).

6 Mak, K. F. *et al*. Measurement of the optical conductivity of graphene. *Phys*. *Rev*. *Lett*. **101**, 196405 (2008).

7 Novoselov, K. S. *et al.* Two-dimensional gas of massless Dirac fermions in graphene. *Nature* **438**, 197-200 (2005).

8 Ariel, V., & Natan, A. Electron effective mass in graphene. http://arxiv.org/abs/1206.6100v2. (2012).

9 Jnawali, G., Rao, Y., Yan, H. & Heinz, T. F. Observation of a transient decrease in terahertz conductivity of single-layer graphene induced by ultrafast optical excitation. *Nano Lett.* **13**, 524-530 (2013)

10 Breusing, M. *et al.* Ultrafast nonequilibrium carrier dynamics in a single graphene layer. *Phys. Rev. B* **83**, 153410 (2011).

11 Sun, D. *et al.* Ultrafast relaxation of excited dirac fermions in epitaxial graphene using optical differential transmission spectroscopy. *Phys. Rev. Lett.* **101**, 157402 (2008).
